# Looking for RED FLAGS: identifying and supporting patients at risk of adverse psychological responses to genetic counselling and testing

**DOI:** 10.1186/1897-4287-10-S2-A17

**Published:** 2012-04-12

**Authors:** S Buscombe

**Affiliations:** 1 The Familial Cancer Centre, Peter McCallum Cancer Institute, Locked Bag 1, A’Beckett Street, Melbourne, Victoria, 8006, Australia

## 

There is a significant body of research indicating having a genetic condition is emotionally burdensome for the individual and their family. One goal of cancer genetic counselling is to assist patients to adapt to the news that they and family members are at significantly increased risk of developing cancer. Numerous studies have shown that the majority of patients who attend cancer genetic services do not report significantly increased long-term psychological distress. However, a small number of patients present with pre-existing complex psychological issues or particular personality traits which are not readily apparent. This subgroup of individuals may experience adverse psychological responses and complex adjustment challenges following their participation in the genetic counselling process.

